# Functional dysbiosis within dental plaque microbiota in cleft lip and palate patients

**DOI:** 10.1186/s40510-019-0265-1

**Published:** 2019-03-25

**Authors:** Kenta Funahashi, Takahiko Shiba, Takayasu Watanabe, Keiko Muramoto, Yasuo Takeuchi, Takuya Ogawa, Yuichi Izumi, Tsutomu Sekizaki, Ichiro Nakagawa, Keiji Moriyama

**Affiliations:** 1 Department of Maxillofacial Orthognathics, Graduate School of Medical and Dental Sciences, Tokyo Medical and Dental University, 1-5-45 Yushima, Bunkyo-ku, Tokyo, 113-8510 Japan; 2 Department of Periodontology, Graduate School of Medical and Dental Sciences, Tokyo Medical and Dental University, 1-5-45 Yushima, Bunkyo-ku, Tokyo, 113-8510 Japan; 3 Department of Chemistry, Nihon University School of Dentistry, 1-8-13 Kanda-Surugadai, Chiyoda-ku, Tokyo, 101-8310 Japan; 4 Research Center for Food Safety, Graduate School of Agricultural and Life Sciences, the University of Tokyo, 1-1-1 Yayoi, Bunkyo-ku, Tokyo, 113-8657 Japan; 5 Department of Microbiology, Graduate School of Medicine, Kyoto University, Yoshida-Konoe-cho, Sakyo-ku, Kyoto, 606-8501 Japan

**Keywords:** Cleft lip and palate, Dental caries, Microbiota, Dysbiosis, Metatranscriptomics

## Abstract

**Background:**

Dental caries is a polymicrobial disease and prevalent among cleft lip and palate (CLP) patients, although their oral hygiene is well maintained. Dysbiosis, the state of imbalance within the dental plaque microbiota, may cause caries prevalence among these patients. However, little is known about how dysbiosis occurs and affects cariogenicity. To find dysbiotic signs, here we conducted a metatranscriptomic analysis for the plaque microbiota in six CLP patients and four controls.

**Methods:**

Total bacterial RNA was extracted from each sample and sequenced. Bacterial composition and functional profiles were estimated from 16S rRNA and mRNA reads, respectively. The mRNA reads were further used for estimating bacterial composition. Species listed in both rRNA-based and mRNA-based bacterial composition were identified as viable taxa with in situ function (VTiF), and the VTiF with a high mRNA-to-rRNA ratio were considered to be transcriptionally active. A network was constructed for each group by connecting two VTiF if their mRNA abundances were positively correlated.

**Results:**

The bacterial composition and functional profiles themselves did not provide remarkable signs of dysbiosis in the CLP group. However, the group-specific active taxa were identified, including streptococcal and *Prevotella* species in the CLP group. Moreover, the network structure was different between groups; *Actinomyces johnsonii* and several species in the CLP group were the active taxa, which were connected based on positive correlations with statistical significance.

**Conclusions:**

Functional dysbiosis within the plaque microbiota was observed such as difference of the network structure between groups, and may be associated with cariogenicity. The observed functional dysbiosis was an invisible change within the microbiota in the oral cavity of CLP patients. This may emphasize the importance of maintaining good oral hygiene of the patients with cleft anomalies.

**Electronic supplementary material:**

The online version of this article (10.1186/s40510-019-0265-1) contains supplementary material, which is available to authorized users.

## Background

Cleft lip and palate (CLP) are congenital anomalies of the oral and maxillofacial regions and occur in 1 to 500 births in Japan [1]. In CLP patients, lack of separation between the oral and nasal cavities by the soft and hard palates is apparent at birth. This anomaly causes esthetic problems and functional disorders such as difficulty in sucking, eating, breathing, and pronouncing words. Palatoplasty effectively solves these problems and enhance the quality of life of CLP patients; however, inflammatory diseases occurring at the surrounding areas of lip and palate such as sinusitis and otitis media are not negligible, even after palatoplasty [2, 3]. In addition, a high prevalence of infectious diseases such as dental caries and gingivitis is indicated in the oral cavity of CLP patients [4–7], although the declining tendency of caries prevalence in CLP patients in a particular nation has been observed recently [8]. A possible reason of the prevalence of these oral diseases is malalignment of the teeth due to the cleft, which leads to low self-cleaning function of the oral cavity and difficulty in maintaining oral hygiene. Despite that CLP patients generally have frequent opportunities to receive oral hygiene instruction and professional teeth cleaning, little is known why caries and gingivitis are prevalent among these patients.

Among several factors such as dietary habits, anatomical structure in the oral cavity, and material properties of tooth, bacteria have been studied as an important factor for caries etiology. Considering that the caries is a polymicrobial disease caused by multiple microbial species in the microbiota [9], dysbiosis would be caused within the microbiota in CLP patients, and would be involved in the prevalence of caries among these patients. Dysbiosis is the state of imbalance in the microbial equilibrium [10], and is associated with oral diseases [11]. *Streptococcus mutans* and lactobacilli are major bacteria associated with caries etiology [9], and exist in great abundance in CLP patients, based on a culture-dependent method [12]. Denaturing gradient gel electrophoresis, based on differences in fragment length, indicated that bacterial diversity was significantly lower in CLP patients than in controls [13]. However, this method only showed differences in a fragment pattern, resulting in insufficient resolution for capturing whole bacterial species in the microbiota.

Dysbiosis is generally used for an imbalance in bacterial composition within the microbiota, whereas an imbalance at the gene transcription level, i.e., functional dysbiosis, is also to be considered. Functional dysbiosis has been reported for the gut microbiota of patients with constipated-irritable bowel syndrome [14], and for the microbiota at the lesion of periodontitis with disease progression [15]. For addressing this issue, comprehensive analysis of bacterial mRNA abundance is widely applied to the microbiota from various environments, and is called metatranscriptomic analysis [16]. This analysis captures the transcriptional activity of every bacterial species in the microbiota, whereas 16S rDNA analysis is used only to determine bacterial composition without regard to their life and death [17].

We previously conducted a metatranscriptomic analysis to compare bacterial activity in periodontitis and peri-implantitis, both of which are oral polymicrobial diseases [18]. In our previous study, the 16S rRNA and mRNA composition between the peri-implantitis and periodontitis samples did not have a clear difference in the microbiota. However, the mRNA-to-rRNA ratio, calculated as the ratio of mRNA abundance to rRNA abundance of each bacterial species, seemed a good index to indicate transcriptional activity. The bacterial species with high mRNA-to-rRNA ratio were different between the two diseases, and such differences within the microbiota may be associated with differences of disease symptoms and prognosis between the two diseases. These analysis ways in our previous study may provide unrecognized bacterial characteristics in CLP patients. In this study, we applied our previous method of metatranscriptomics to dental plaque microbiota in CLP and non-CLP patients, in order to examine whether functional dysbiosis was present within plaque microbiota in CLP patients.

## Methods

### Patient selection and clinical assessment

From 2012 to 2014, CLP and non-CLP patients undergoing orthodontic treatment at the Tokyo Medical and Dental University Hospital Faculty of Dentistry were considered for this study. Six nonsyndromic CLP patients (i.e., CLP group) and four non-CLP patients (i.e., control group) were finally enrolled (Table 1), because a sufficient amount of samples was available from only these ten patients who provided informed consent. All CLP patients were treated with plastic surgery of the lip and palate during young childhood. All individuals did not use multibracket appliances, but the use of any other fixed and removable appliances was not taken into account for selection of patients. All individuals were not treated with antimicrobial agents within 3 months before sample collection. Crowding of teeth was assessed if either maxillary or mandibular dental arch had at least 5 mm of crowding [19]. The following clinical parameters were assessed: oral hygiene index (OHI) [20], gingival index (GI) [21], and the number of decayed, missing, and filled teeth (DMFT) for all deciduous and permanent dentition [22].

**Table 1:** Clinical characteristics of the study participants

Sample name	Disease type	Sex	Age (year)	Appliance	OHI	GI	DMFT	Crowding	Ectopic, impacted, or agenetic teeth
CLP1	Unilateral CLP	M	12	No appliance	1.7	0.0	1	−	+
CLP2	Unilateral CLP	F	9	Quad-helix appliance	1.2	0.0	0	+	+
CLP3	Unilateral CLP	F	12	Retainer	1.0	0.0	0	+	+
CLP4	Bilateral CLP	M	15	Lingual arch	1.3	0.0	2	+	+
CLP5	Unilateral CLP	F	7	Quad-helix appliance	1.0	0.3	0	+	−
CLP6	Unilateral CLP	M	12	Palatal obturator	1.0	0.2	1	−	+
C1	Control	F	10	No appliance	1.0	0.0	0	−	−
C2	Control	F	10	Headgear	0.8	0.0	0	+	−
C3	Control	M	11	Expansion plate with tongue crib	0.8	0.3	0	+	−
C4	Control	M	8	Maxillary protractive appliance	1.3	0.0	5	−	−

### Procedure for obtaining the Illumina sequence data

For each individual, a supragingival plaque sample was collected from all surfaces of all teeth by using a sterilized toothpick. Professional tooth cleaning was stopped for 1 month, and patients were instructed to stop their own plaque control at least 1 h before the sample collection. The collected plaque was placed into PM1 buffer in the PowerMicrobiome RNA Isolation kit (MO BIO Laboratories, Carlsbad, CA, USA) in a sterile tube, and was stored at − 80 °C. The RNA extraction, cDNA synthesis, library preparation, and Illumina sequencing were conducted, as described previously [18] with the following modifications. The SuperScript® Double-Stranded cDNA Synthesis kit (Thermo Fisher Scientific, Waltham, MA, USA) and 6-mer random primer were used instead of the SMARTer Ultra Low RNA kit (Clontech, Mountain View, CA, USA). MiSeq (Illumina, Inc., San Diego, CA, USA) reads were generated as 300-bp paired-end.

### Data analysis

The preprocessing of the Illumina reads and all data analysis were conducted, as described previously [18]. In brief, 16S rRNA reads were reconstructed to form OTUs, and the OTUs were taxonomically assigned based on the Human Oral Microbiome Database (HOMD; v14.5). On the other hand, mRNA reads were assigned based on the National Center for Biotechnology Information Non-redundant Protein Database (NCBI nr; as of January 10, 2017), Virulence Factors Database (VFDB; as of December 23, 2016), and Microbial Virulence Database (MvirDB; as of December 20, 2016). The species taxa detected in both 16S rRNA and mRNA were considered “viable taxa with *in situ* function (VTiF)”, and the VTiF with a high mRNA-to-rRNA ratio as log2 values of ≥ 6 were considered “active taxa.” The two VTiF with a positive correlation in mRNA abundance with the Strong, Prosperous And Resilient Communities Challenge (SparCC) values ≥ 0.995 were connected to construct a network structure for each group. In the network, the taxa which were detected in more than half of patients and connected by a positive correlation with a statistical significance were considered “interacting core taxa”. Mann-Whitney *U* test was used for testing statistical significance of difference between two independent groups in this study, instead of Wilcoxon’s signed-rank test [18].

## Results

### Clinical characteristics and summary of the sequence reads

The CLP group consisted of four left, one right, and one bilateral CLP subjects, and the participants used removable or fixed orthodontic appliances except for one CLP and one control subject (Table 1). Crowding of teeth was present in six participants (four CLP and two control subjects), and five participants had ectopic, impacted, or agenetic teeth (five CLP subjects). The average age was 11.2 years (range, 7–15 years). OHI ranged between 0.8 and 1.7. GI was scored to be 0 in four CLP subjects and three control subjects, whereas GI greater than 0 was scored in two CLP subjects and one control subject. DMFT were scored to be 0 in three CLP subjects and three control subjects, whereas DMFT greater than 0 was scored in three CLP subjects and one control subject. No significant differences between the CLP and control groups were observed in the age and the clinical parameters. The total number of preprocessed reads in each subject ranged between 3,461,365 and 6,603,648 (average number, 4,828,088) among 10 participants (Additional file 1: Table S1). The number of preprocessed reads was not significantly different between the groups.

### Bacterial composition estimated from the 16S rRNA reads

Preprocessed reads in metatranscriptomic analysis generally contain rRNA, mRNA, and other miscellaneous RNA reads. The 16S rRNA reads in our data were used to estimate the bacterial composition. From the 16S rRNA reads ranging between 57,498 and 405,306 (3.6–16.7% preprocessed reads; Additional file 1: Table S1), the operational taxonomic units (OTUs) were formed to be 62.2 ± 15.7 in the CLP group and 62.3 ± 8.5 in the control group. These OTUs were thereafter considered reconstructed 16S rRNAs (rc-rRNAs). No significant difference existed between the two groups in the number of OTUs (*P* = 0.37) and Shannon’s diversity index (*P* = 0.27), which was used to estimate the diversity of bacterial composition with the distribution of bacterial abundances (Additional file 1: Figure S1). The rarefaction curve showed the saturation in the number of OTUs for each individual (Additional file 1: Figure S1).

**Fig. 1:**
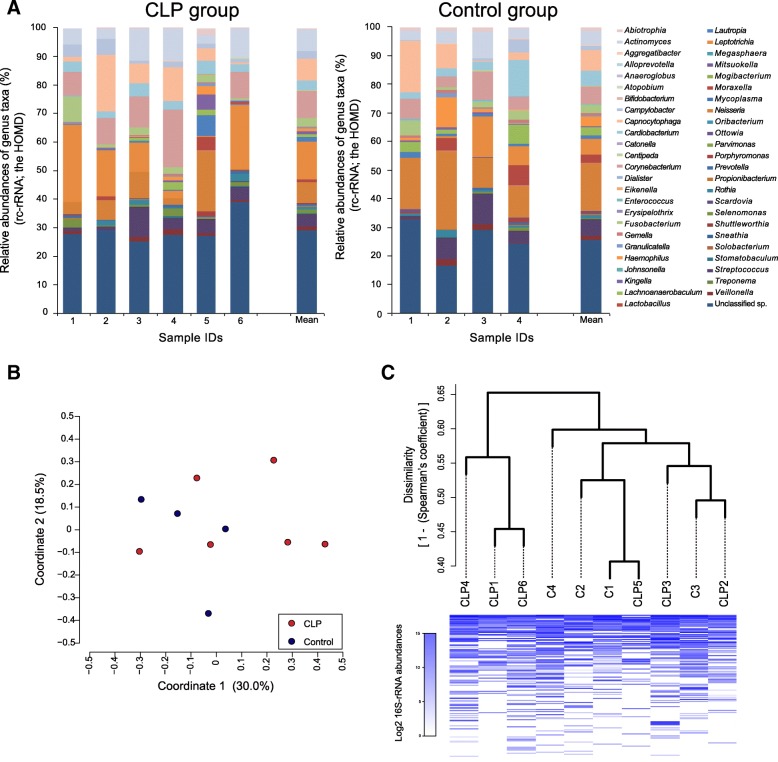
Bacterial composition estimated from 16S rRNA reads. **a** The bar charts present the bacterial composition assigned with the HOMD at the genus level, shown as the relative abundance. Each color corresponds to the genus indicated on the right side. **b** First coordinates in the PCoA for the bacterial composition assigned with the HOMD are plotted with second coordinates in the scatter graph. The CLP and control participants are indicated by red and blue, respectively. **c** The dissimilarity matrix is derived from Spearman’s rank correlation coefficient between any two participants and is used to construct a dendrogram of hierarchical clustering. A heat map of log2 rc-rRNA abundances is shown below the dendrogram

The rc-rRNAs of 70.7 ± 4.9% in the CLP group and 74.2 ± 7.1% in the control group were assigned 140 bacterial species in 49 genera based on the HOMD (Fig. 1a, Additional file 1: Table S2). Although *Leptotrichia* in the CLP group and *Neisseria* in the control group were the most predominant genera on average (13.2% and 16.8%, respectively), these genera were also predominantly observed in the counterpart group (Additional file 1: Table S2). Seven genera in the CLP group and two genera in the control group were quite low in abundance but were specific to each group, and consisted of the following species: *Anaeroglobus geminatus*, *Bifidobacterium dentium*, *Lactobacillus vaginalis*, *Lactobacillus fermentum*, *Lactobacillus rhamnosus*, *Mycoplasma salivarium*, *Scardovia wiggsiae*, *Shuttleworthia satelles*, and *Sneathia amnii* in the CLP group; and *Erysipelothrix tonsillarum* and *Mitsuokella multacida* in the control group (Additional file 1: Table S2). In the principal coordinate analysis (PCoA) plots and hierarchical clustering dendrograms, the bacterial composition was not clearly dissimilar between groups and was rather dispersed across groups (Fig. 1b, c), supported by the analysis of similarity (ANOSIM; *R* = 0.16 and *P* = 0.10). The samples were not clustered according to presence and absence of appliances and their removability (Additional file 1: Figure S2).

### Functional profiles estimated from the mRNA reads by using an analysis pipeline

**Fig. 2:**
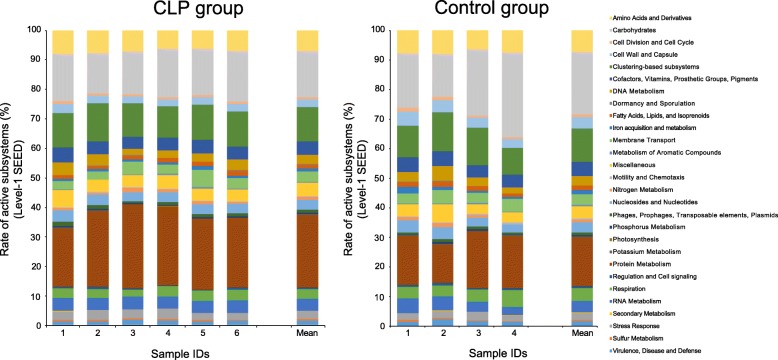
Functional profiles estimated from mRNA reads with the SEED. The bar charts present gene functions as relative abundances. Each color corresponds to the functions in the level-1 SEED subsystem, indicated on the right side

Using the Metagenomics Rapid Annotation using Subsystem Technology (MG-RAST) analysis pipeline, the mRNA reads ranging between 422,111 and 966,679 in number (11.8–22.4% of preprocessed reads; Additional file 1: Table S1) were assigned 10,255 ± 4895.8 gene functions in the CLP group and 13,325 ± 15,142 functions in the control group based on the level-4 SEED subsystem (i.e., the subsystem at each function level) in the SEED database. The functional profiles assigned with the SEED database were similar among individuals across groups (Fig. 2), and the similarity was supported by the ANOSIM (*R* = 0.39 and *P* = 0.024). Protein metabolism, carbohydrates, and clustering-based subsystems (i.e., the genes functionally coupled but with unknown function) in the level-1 SEED subsystem (i.e., the subsystem at the top level) were predominant in every individual (Additional file 1: Table S3).

On the other hand, by using the MG-RAST, the mRNA reads were assigned 9830 ± 4568.4 gene functions in the CLP group and 11,457 ± 12,269 functions in the control group based on the Kyoto Encyclopedia of Genes and Genomes (KEGG) database. The rank order of functions from the top to several tens was nearly the same with slight differences, and ≥ 99% of functions in abundance was common between the groups (Additional file 1: Table S4). At functional level-1 (i.e., the subsystem at the top level), metabolism was the most predominant (43.3% in the CLP group and 44.7% in the control group in abundance), followed by genetic information processing (28.0% in the CLP group and 28.3% in the control group) and environmental information processing (16.1% in the CLP group and 14.4% in the control group) (Additional file 1: Table S4). Both assignments using the SEED and KEGG databases were not significantly different in the functional profiles between the groups in the ANOSIM (SEED database: *R* = 0.39 and *P* = 0.024; KEGG database: *R* = 0.15 and *P* = 0.16). The samples did not show remarkable similarity of the functional profiles according to presence and absence of appliances and their removability. Further profiling with other several databases is described in Additional file 1.

### Bacterial composition estimated from the mRNA reads

**Fig. 3:**
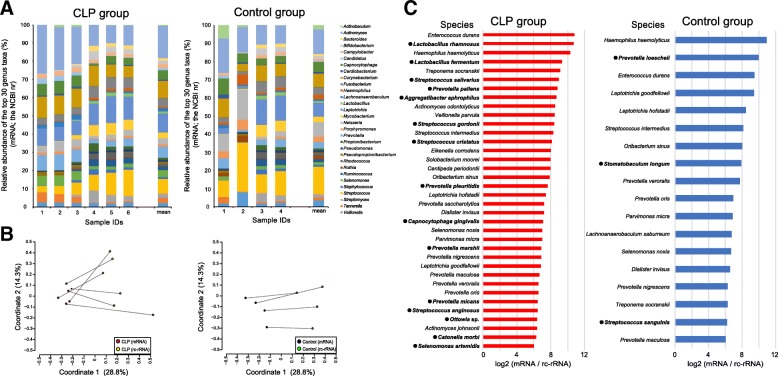
Bacterial composition estimated from mRNA reads and comparative analysis of 16S rRNA-based and mRNA-based profiles. **a** In the bar charts, bacterial composition assigned using the NCBI nr at the genus level is presented as the relative abundance. Each color corresponds to a genus on the right side. **b** For the bacterial composition assigned with the HOMD for rc-rRNA and NCBI nr for mRNA reads, first coordinates in the PCoA are plotted with second coordinates in scatter graphs. For CLP subjects (on the left side), the rc-rRNA-based and mRNA-based compositions are indicated by red and yellow, respectively. For the control participants (on the right side), the rc-rRNA-based and mRNA-based compositions are indicated by blue and green, respectively. **c** The bar charts show the mean log2 ratio of the active taxa, defined as taxa with a log2 mRNA-to-rRNA ratio of ≥ 6. Group-specific active taxa are indicated by bold characters and filled circles (on the left side of names)

The mRNA reads were taxonomically assigned using the NCBI nr, which describes the taxonomic origin of each gene. The taxonomically unclassified reads were quite few (on average, 1.1 ± 0.80% in the CLP group and 1.1 ± 1.0% in the control group). The remaining reads were classified at the species level (Additional file 1: Table S5). Bacterial composition between groups was not clearly different (*R* = − 0.0026 and *P* = 0.41, based on the ANOSIM), and the top 30 genera were similar across groups (Fig. 3a). In both groups, the most predominant genus was *Actinomyces* (on average, 14.0% in the CLP group and 10.6% in the control group). The rank distribution was different between the groups from the top to several tens; however, most genera in this rank range were detected in both groups. At the species level, *Corynebacterium matruchotii* and *Leptotrichia hofstadii* were the most predominant in the CLP group (4.9 ± 0.80%) and in the control group (4.3 ± 4.1%), respectively. These species ranked second in the counterpart group (Additional file 1: Table S5). Similar to the bacterial composition estimated from 16S rRNA reads, the samples were not clustered according to presence and absence of appliances and their removability (Additional file 1: Figure S2).

### Calculation of the mRNA-to-rRNA ratio to identify active taxa

The bacterial composition estimated from the mRNA reads differed from the composition estimated from the rc-rRNA (*R* = 0.82 and *P* = 0.003 in the CLP group, and *R* = 0.43 and *P* = 0.061 in the control group, based on the ANOSIM; Fig. 3b). Among the bacterial species estimated from the rc-rRNA and mRNA analyses, 114 species in the CLP group and 101 species in the control group were detected in both rc-rRNA and mRNA, and were thus identified as VTiF (Additional file 1: Table S6). Eighty-nine VTiF were common between the groups, whereas 25 VTiF in the CLP group and 12 VTiF in the control group were group-specific. We then calculated the mRNA-to-rRNA ratio for each VTiF, and classified them as active taxa if log2 values of the ratio were ≥ 6 (Additional file 1: Table S6). Thirty-six active taxa in the CLP group and 18 active taxa in the control group were identified (Fig. 3c). Among them, 15 taxa in the CLP group and 3 taxa in the control group were group-specific (Fig. 3c), as follows: *Lactobacillus rhamnosus*, *Lactobacillus fermentum*, *Streptococcus salivarius*, *Prevotella pallens*, *Aggregatibacter aphrophilus*, *Streptococcus gordonii*, *Streptococcus cristatus*, *Prevotella pleuritidis*, *Capnocytophaga gingivalis*, *Prevotella marshii*, *Prevotella micans*, *Streptococcus anginosus*, *Ottowia* sp., *Catonella morbi* and *Selenomonas artemidis* in the CLP group, and *Prevotella loescheii*, *Stomatobaculum longum*, and *Streptococcus sanguinis* in the control group.

### Networks estimated from the positive correlation of mRNA abundances, and the identification of interacting core taxa

**Fig. 4:**
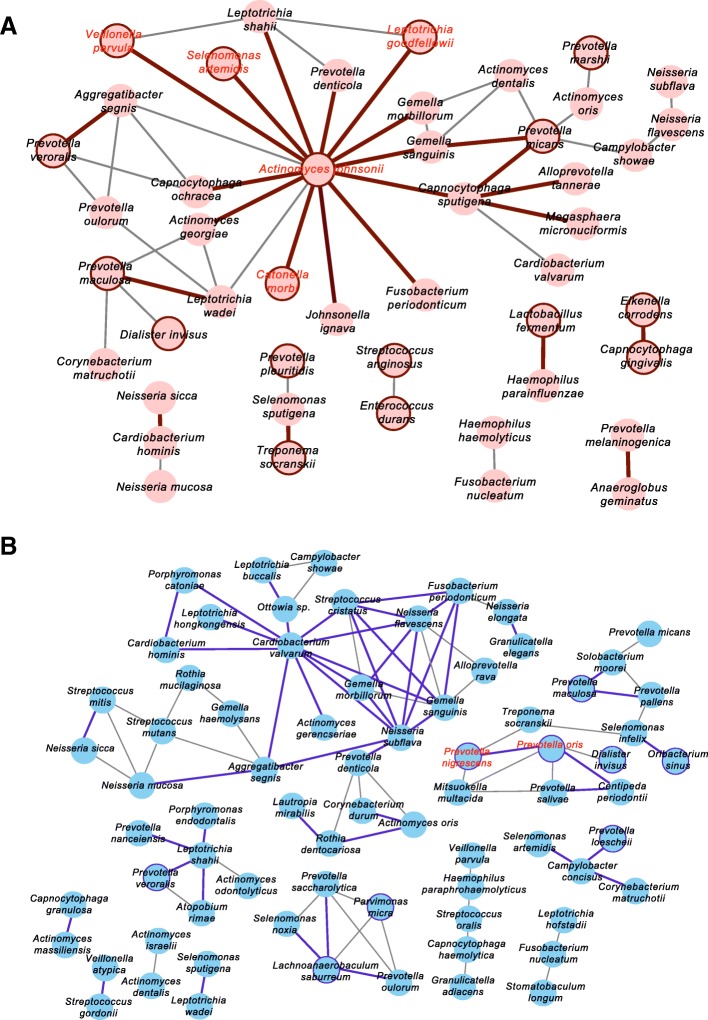
Network estimated from the correlation of mRNA abundance and interacting core taxa. Two VTiF are connected if their mRNA abundances are positively correlated with a value of SparCC ≥ 0.995, for **a** the CLP group and **b** the control group. Active taxa are indicated by bold circles, and pairs of active taxa with a significant positive correlation are indicated by bold lines. Interacting core taxa (i.e., pairs of active taxa with a significant positive correlation) are indicated by red characters

For each pair of two VTiF in each group, the correlation coefficient was calculated from the mRNA abundance of all individuals by using SparCC, and was used for constructing the network structure for each group. Forty-seven of 114 species in the CLP group and 73 of 101 species in the control group participated as the nodes in the networks (Fig. 4). The VTiF were connected with 1.1 edges in the CLP group and 1.3 edges in the control group per node. The value of clustering coefficient, an indicator of the tendency that the nodes are clustered together, was 0.18 in the CLP group and 0.35 in the control group.

In the networks, the active taxa were included with the following numbers: 17 of 36 in the CLP group and 8 of 18 in the control group (Fig. 4). The species pairs with a significant positive correlation in the CLP group (23 pairs) were nearly one-half of that in the control group (48 pairs); however, the pairs of active taxa with a significant positive correlation (i.e., the interactive core taxa) were more abundant in the CLP group (e.g., four pairs formed by five interactive core taxa) than in the control group (e.g., one pair formed by two interactive core taxa) (Fig. 4, Additional file 1: Figure S3).

## Discussion

For medical clinicians, dysbiosis within gut microbiota is a key problem in intestinal health because it contributes to various adverse effects such as altered immune responses [23], constipation [24], and autoimmune diseases [25]. The oral cavity is also affected by dysbiosis within the microbiota as a form of infectious diseases including caries and periodontitis [11]. We examined the presence of dysbiosis within dental plaque microbiota in the CLP group by profiling the bacterial composition, based on rc-rRNA. Although dead bacteria were excluded from our analysis for rc-rRNA, the presence of dysbiosis was unclear in the CLP group (Fig. 1). Some caries-related bacterial species such as *Lactobacillus vaginalis* [26] and *Scardovia wiggsiae* [27], which were specifically detected in the CLP group, were one of the few characteristics to be described in the analysis. These species reportedly have a role in childhood caries [26, 27]. In addition, *S*. *wiggsiae* was reported to be a potential caries pathogen owing to its tolerance to acidic environments and its ability to produce acid, regardless of the presence or absence of *S*. *mutans* [27]. Considering the group-specificity of *L*. *vaginalis* and *S*. *wiggsiae* under the situation that caries prevalence of both groups was similarly low, these species may be associated with caries etiology in CLP patients with respect to their presence in disease sites.

We then analyzed the functional profiles estimated from mRNA to predict if there were any signs of functional dysbiosis in the CLP group, as in previous studies focusing on caries and periodontitis [28] or focusing on periodontitis [29]. These studies reported a significantly high abundance in particular functions in controls or in patients. However, despite that a database with comprehensive broad functions (i.e., NCBI nr), databases in which the functions were limited to virulence-related functions (i.e., VFDB and MvirDB) and an analysis pipeline (i.e., MG-RAST) were used for the search, our data of the functional profiles themselves were thoroughly less indicative of dysbiosis as an imbalance in transcriptional activities (Fig. 2, Additional file 1: Figure S4). This finding was similar to our previous report for peri-implantitis and periodontitis [18]. Even glucosyltransferases, which have been reported as promising targets for preventing caries with inhibitory molecules rather than eradicating cariogenic bacteria [30], were not characteristic because they were not predominant in and not specific to either group.

In contrast to these analyses, the prediction of the bacterial composition from the mRNA reads led to observation of remarkable differences between the CLP and control groups in the members of the VTiF. Although the bacterial composition itself estimated from mRNA was not obviously different between groups, the mRNA-to-rRNA ratio was an objective index of transcriptional activity of each bacterial species (Fig. 3). The active taxa specific to the CLP group included several streptococcal and *Prevotella* species associated with caries (Fig. 3c). These active taxa, i.e., species *Streptococcus salivarius*, *Streptococcus gordonii*, and *Streptococcus cristatus*, and the genus *Prevotella*, were highly abundant in patients with caries in previous studies [31–33]. These species would cause a dysbiotic state in the plaque microbiota by being abundant and exhibiting high transcriptional activities, although their functional traits could not be obtained in this study. The enrichment of public databases would improve the functional assignment of genes currently assigned to encode hypothetical proteins.

Furthermore, the network analysis provided another viewpoint for functional activity (Fig. 4). In addition to the observed differences in the number of species involved in the networks and the overall form of networks between the groups, the lower value of the clustering coefficient in the CLP group than in the control group suggested that the network was somewhat more fragile in the CLP group than in the control group [34]. Among species in the networks, *Actinomyces johnsonii* was in the center of the main network in the CLP group and was a member of the interacting core taxa in the CLP group (Fig. 4). *A*. *johnsonii* was originally classified as the species *Actinomyces naeslundii* before its reclassification in the current species taxon [35], and may be associated with caries on root surfaces by preserving the pH homeostasis [36]. It is possible that *A*. *johnsonii* caused functional dysbiosis within the plaque microbiota, like a keystone species that causes a dysbiotic state despite its low abundance [37]. The network would thus be less robust in the CLP group than in the control group, but functional communication among *A*. *johnsonii* and other species in the network may increase the virulence of the microbiota.

## Conclusions

Although a variety of dietary, anatomical, and other non-bacterial factors among participants was not considered, we observed an aspect of functional dysbiosis within the plaque microbiota as the varied mRNA-to-rRNA ratio in CLP patients by conducting metatranscriptomic analysis. We found that the mRNA-to-rRNA ratio could indeed be used to indicate transcriptional activity of each bacterial taxon, but it should be considered that rRNA abundance can be high in the resting, dormant, or inactive cells [38], although the actually active cells would occupy a large fraction of the microbiota.

The findings in this study will provide clues to understanding etiology of caries as a polymicrobial disease, and the analysis ways of identifying active taxa and constructing networks from rRNA and mRNA abundance would be useful for other polymicrobial diseases in the oral cavity and other body sites. Further studies are needed to find how dysbiosis occurs within the microbiota if caries prevalence is high, and to clarify the influence of nasal microbiota on dysbiosis within the oral and plaque microbiota.

In addition, future studies will include more samples to reduce the potential effect of individuality of the microbiota on the comparison between groups, which would lead to finding unknown differences of the microbiota between groups as a kind of functional dysbiosis. Moreover, this study highlighted functional dysbiosis of the oral microbiota in CLP patients as an invisible change in the oral cavity, in contrast to visible morphological and functional disorders in the oral cavity. To understand the presence of dysbiosis within the oral microbiota would be important for clinicians treating CLP patients. The clinicians would make more efforts to maintain good oral hygiene of CLP patients by considering techniques and frequency of professional oral care and improving plaque control skills of patients themselves and their families.

## Additional file

Additional file 1: **Figure S1.** Number of OTUs, Shannon’s diversity index, and rarefaction curves. **Figure S2.** PCoA plots with information of appliances. **Figure S3.** Scatter graphs of mRNA abundances for pairs of interacting core taxa. **Figure S4.** PCoA plots for the functional profiles assigned using BLASTX. **Table S1.** Summary of Illumina MiSeq reads and the derived data. **Table S2.** Rank distribution of species assigned for the rc-rRNA OTUs (relative abundance per participant). **Table S3.** Rank distribution of the level-1 SEED subsystem functions assigned for the mRNA reads (relative abundance per participant). **Table S4.** Rank distribution of the level-2 KEGG functions assigned for the mRNA reads (relative abundance per participant). **Table S5.** Rank distribution of the top 50 species assigned for the mRNA reads (relative abundance per participant). **Table S6.** Rank distribution of VTiF (mean RPKM values among the participants and the mRNA-to-rRNA ratio). **Table S7.** Rank distribution of the top 50 VFDB functions assigned for the mRNA reads (relative abundance per participant). **Table S8.** Rank distribution of the top 50 MvirDB functions assigned for the mRNA reads (relative abundance per participant). (PDF 454 kb)

## Abbreviations

ANOSIM: Analysis of similarity
CLP: Cleft lip and palate
DMFT: Decayed, missing, and filled teeth
GI: Gingival index
HOMD: Human Oral Microbiome Database
KEGG: Kyoto Encyclopedia of Genes and Genomes
MG-RAST: Metagenomics Rapid Annotation using Subsystem Technology
MvirDB: Microbial Virulence Database
NCBI nr: National Center for Biotechnology Information Non-redundant Protein Database
OHI: Oral hygiene index
OTU: Operational taxonomic unit
PCoA: Principal coordinate analysis
rc-rRNA: Reconstructed 16S rRNA
SparCC: Strong, Prosperous and Resilient Communities Challenge
VFDB: Virulence Factors Database
VTiF: Viable taxa with in situ function
