# Supplementary material for: Functional dysbiosis within dental plaque microbiota in cleft lip and palate patients
Source: Prog Orthod. 2019 Mar 25;20:11. doi: 10.1186/s40510-019-0265-1 (PMC6431681; doi:10.1186/s40510-019-0265-1)
Supplement: Supplementary file 1 — Figure S1. Number of OTUs, Shannon’s diversity index, and rarefaction curves. Figure S2. PCoA plots with information of appliances. Figure S3. Scatter graphs of mRNA abundances for pairs of interacting core taxa. Figure S4. PCoA plots for the functional profiles assigned using BLASTX. Table S1. Summary of Illumina MiSeq reads and the derived data. Table S2. Rank distribution of species assigned for the rc-rRNA OTUs (relative abundance per participant). Table S3. Rank distribution of the level-1 SEED subsystem functions assigned for the mRNA reads (relative abundance per participant). Table S4. Rank distribution of the level-2 KEGG functions assigned for the mRNA reads (relative abundance per participant). Table S5. Rank distribution of the top 50 species assigned for the mRNA reads (relative abundance per participant). Table S6. Rank distribution of VTiF (mean RPKM values among the participants and the mRNA-to-rRNA ratio). Table S7. Rank distribution of the top 50 VFDB functions assigned for the mRNA reads (relative abundance per participant). Table S8. Rank distribution of the top 50 MvirDB functions assigned for the mRNA reads (relative abundance per participant). (PDF 454 kb) [file 40510_2019_265_MOESM1_ESM.pdf]

Functional dysbiosis within dental plaque microbiota in cleft lip and palate patients

K. Funahashi, T. Shiba, T. Watanabe, K. Muramoto, Y. Takeuchi, T. Ogawa, Y. Izumi, T. Sekizaki, I. Nakagawa and K. Moriyama

Supplementary Material

Supplementary Results

Functional profiles estimated from mRNA reads using BLASTX and various databases

In addition to conducting functional assignment by using the MG-RAST, the mRNA reads were further assigned by using the NCBI nr and two databases of virulence genes (i.e., VFDB and MvirDB) using BLASTX. Before the assignment, all reads were categorized into 210,290 ± 78,421 clusters, and the clusters derived from 16S rRNA genes were removed to obtain 175,144 ± 80,587 clusters of mRNA reads (Table S1). The mRNA reads were assigned 37,706 ± 7,014 gene functions in the CLP group and 39,279 ± 15,047 functions in the control group based on the NCBI nr. The genes encoding hypothetical protein in the NCBI nr accounted for nearly one-half of all functions in abundance in each group (47.2% in the CLP group and 47.4% in the control group). The group-specific functions were 3.5% in the CLP group and 4.9% in the control group. The most predominant gene encoding a protein with known function among the group-specific functions was only 0.038% in the CLP group (for RNA-dependent RNA polymerase) and 0.012% in the control group (for sanguinicin K11 precursor protein). The functional profiles assigned using the NCBI nr were not significantly different between the groups ($R = -0.075$ and $P = 0.58$, based on the ANOSIM; Figure S4).

The mRNA reads were assigned 1,234 ± 179.3 gene functions in the CLP group and 1,284 ± 475.4 functions in the control group based on the VFDB, and 1,300 ± 157.7 functions in the CLP group and 1,342 ± 434.5 functions in the control group based on the MvirDB (Table S1). The elongation factor, surface-anchored fimbrial subunit, glyceraldehyde-3-phosphate dehydrogenase, and phosphopyruvate hydratase in the VFDB were predominant and were nearly 20% in abundance in both groups (Table S7). The group-specific functions in the VFDB were quite few (2.4% in the CLP group and 2.8% in the control group). The most predominant function among the group-specific functions was only 0.075% in the CLP group (for fimbrial protein P9-2 pilin) and 0.079% in the control group (for manganese ABC transporter substrate-binding protein). In the MvirDB, the translation initiation factor, 50S ribosomal protein, pyruvate formate lyase, ATP-binding protein, and surface-anchored fimbrial subunit were predominant and accounted for more than 10% in both groups (Table S8). As in the VFDB, the group-specific functions in the MvirDB were quite few (2.3% in the CLP group and 2.5% in the control group). The most predominant function among the group-specific functions was only 0.052% in the CLP group (for transcription antitermination factor) and 0.285% in the control group (for type IV pilin). The functional profiles assigned based on the VFDB and MvirDB were not significantly different between the groups (VFDB: $R = -0.053$ and $P = 0.56$; MvirDB: $R = 0.16$ and $P = 0.10$, based on the ANOSIM; Figure S4).

Supplementary Figures

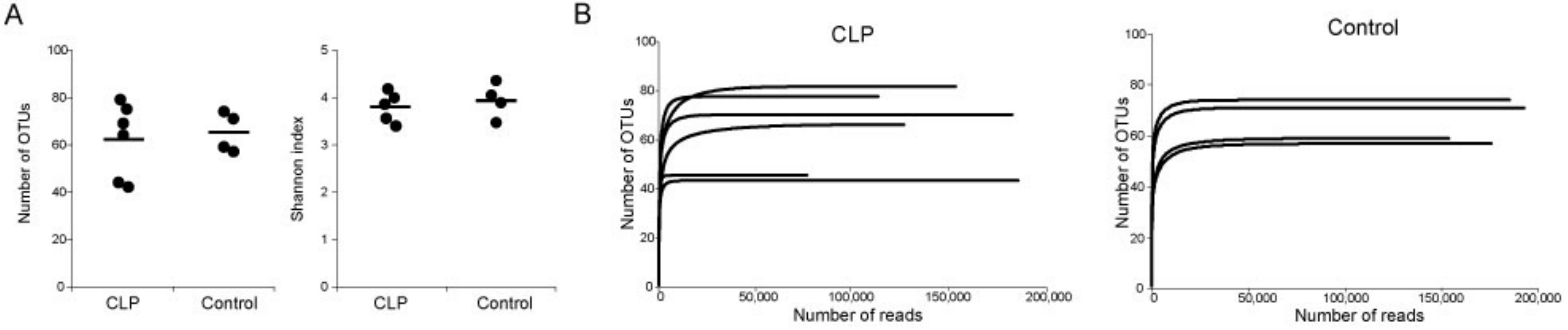

Figure S1. Number of OTUs, Shannon's diversity index, and rarefaction curves. (A) The number of OTUs and Shannon's diversity index for each participant are shown in the scatter graphs. The horizontal bars indicate the mean values among the participants. (B) A rarefaction curve for each participant is shown with the range of horizontal axis from 0 to the number of reads in the corresponding participant.

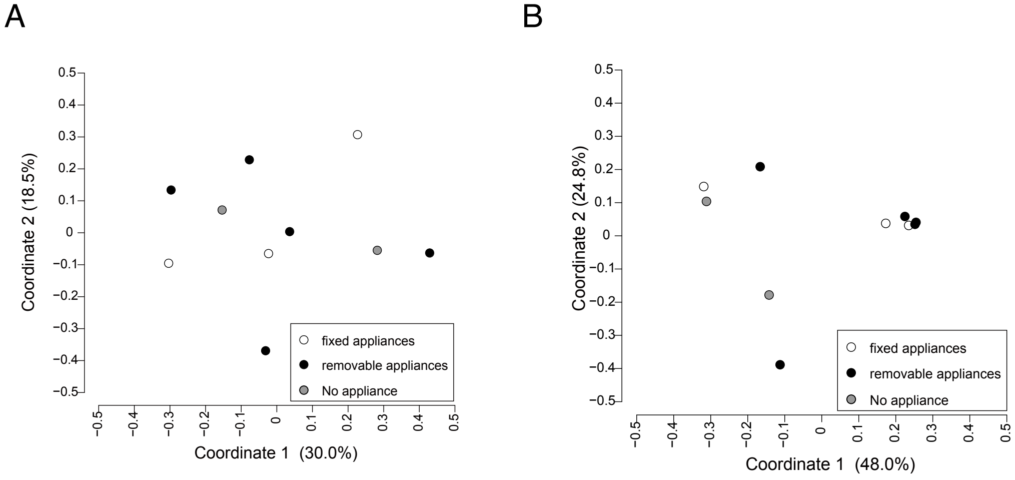

Figure S2. PCoA plots with information of appliances. The bacterial composition estimated from 16S rRNA and mRNA reads are shown in (A) and (B), respectively. Participants with fixed and removable appliances are indicated by open and filled circles, respectively, and participants without any appliances are indicated by gray circles.

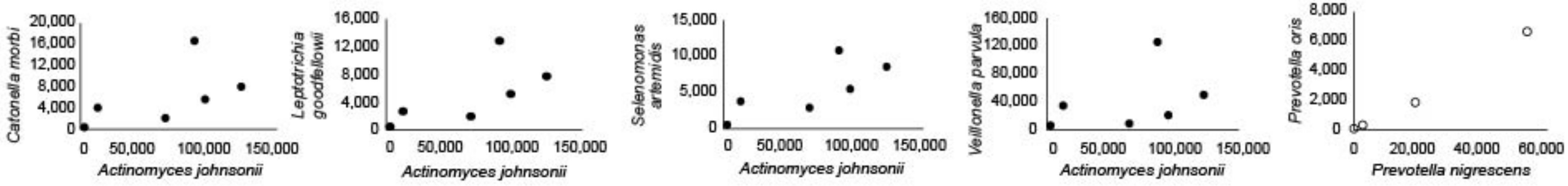

Figure S3. Scatter graphs of mRNA abundances for pairs of interacting core taxa. The CLP patients and control participants are indicated by filled and open circles, respectively.

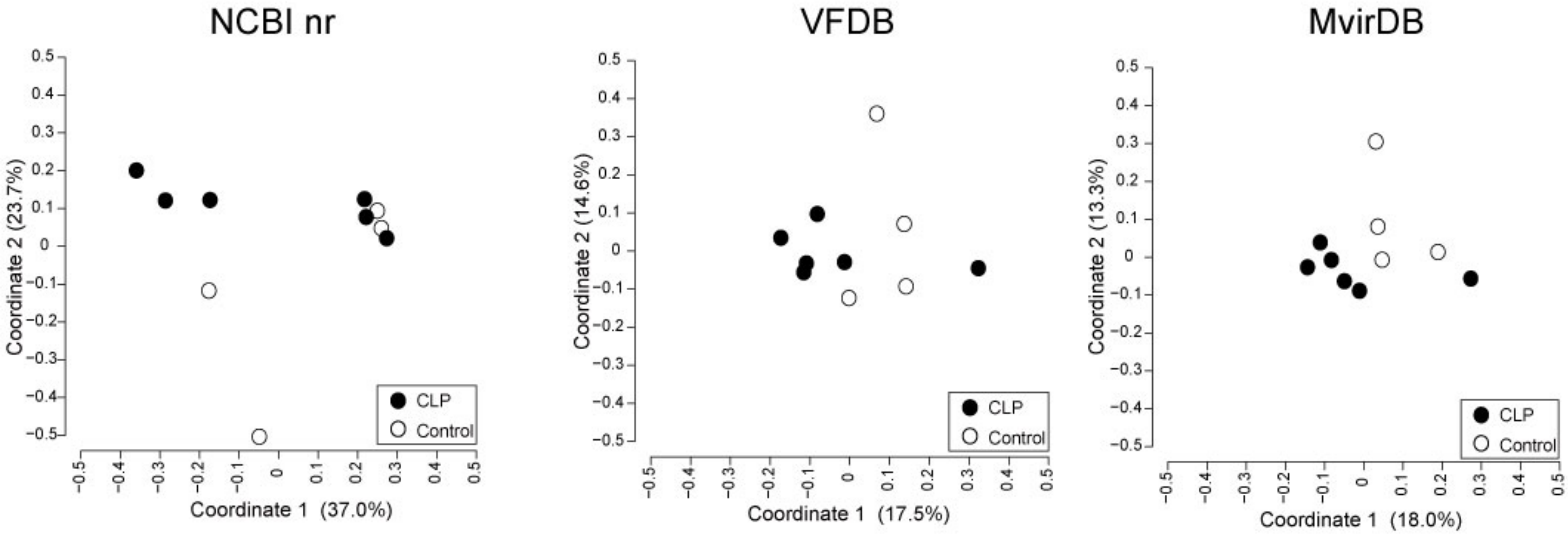

Figure S4. PCoA plots for the functional profiles assigned using BLASTX. First coordinates in PCoA for the functional profiles assigned by using the NCBI nr, VFDB, and MvirDB are plotted with second coordinates in the scatter graphs. The CLP patients and control participants are indicated by filled and open circles, respectively.

Supplementary Tables

Table S1. Summary of Illumina MiSeq reads and the derived data.

Sample name	Number of reads					Number of formed clusters		Number of assigned functions				
	Raw	Preprocessed		EMIRGE	MG-RAST	All	mRNA	SEED	KEGG	NCBI nr	VFDB	MvirDB
		Paired	Unpaired									
CLP1	3,886,942	3,241,544	219,821	57,498	774,136	284,914	243,230	17,421	16,302	36,508	1,367	1,412
CLP2	5,932,626	5,005,270	351,103	405,306	747,449	191,697	137,170	7,479	7,527	28,536	1,094	1,168
CLP3	5,398,948	4,530,979	385,671	277,902	703,790	184,543	167,892	8,026	7,837	32,733	1,212	1,301
CLP4	7,054,912	5,876,240	510,428	350,420	966,679	283,627	262,607	14,279	13,601	37,474	1,486	1,498
CLP5	4,051,374	3,131,673	446,209	130,207	422,111	102,720	53,982	3,961	3,594	42,805	991	1,070
CLP6	4,447,902	3,360,050	523,921	138,199	669,497	198,626	168,842	10,364	10,117	48,177	1,255	1,348
C1	4,960,774	4,136,023	277,602	346,092	583,074	145,333	91,835	4,090	3,839	17,905	925	988
C2	5,233,586	4,449,558	345,749	254,016	992,930	367,024	323,040	35,898	29,717	52,605	1,969	1,956
C3	7,210,598	6,161,642	442,006	273,893	788,363	172,714	153,670	5,239	4,818	40,814	1,006	1,089
C4	5,514,144	4,309,251	576,140	160,140	704,580	171,697	149,170	8,072	7,452	45,791	1,234	1,333

Table S2. Rank distribution of species assigned for the rc-rRNA OTUs (relative abundance per participant).

CLP				Control			
Species	Group-specificity	Mean (%)	Standard deviation	Species	Group-specificity	Mean (%)	Standard deviation
Corynebacterium matruchotii	Common	9.041	6.154	*Neisseria sicca*	Common	9.613	12.153
Leptotrichia wadei	Common	5.514	3.757	*Corynebacterium matruchotii*	Common	5.188	3.486
Capnocytophaga granulosa	Common	5.493	7.438	*Cardiobacterium hominis*	Common	5.091	5.108
Leptotrichia buccalis	Common	4.526	4.838	*Capnocytophaga granulosa*	Common	4.987	8.101
Streptococcus sanguinis	Common	2.909	3.476	*Leptotrichia buccalis*	Common	3.826	5.293
Cardiobacterium hominis	Common	2.891	1.692	*Neisseria flavescens*	Common	3.275	6.549
Fusobacterium nucleatum	Common	2.724	3.153	*Haemophilus parainfluenzae*	Common	3.208	4.916
Neisseria sicca	Common	2.152	3.334	*Lachnoanaerobaculum umeaense*	Common	2.823	2.760
Actinomyces naeslundii	Common	2.002	2.582	*Moraxella catarrhalis*	Common	2.802	3.456
Neisseria oralis	Common	1.993	4.251	*Fusobacterium nucleatum*	Common	2.320	2.058
Campylobacter showae	Common	1.818	2.305	*Actinomyces naeslundii*	Common	2.212	1.629
Leptotrichia hofstadii	Common	1.756	3.177	*Streptococcus oralis*	Specific	2.146	2.741
Neisseria mucosa	Common	1.726	4.191	*Streptococcus mitis*	Common	1.221	1.623
Actinomyces massiliensis	Common	1.560	0.827	*Neisseria subflava*	Common	1.189	1.427
Lautropia mirabilis	Common	1.510	2.941	*Campylobacter showae*	Common	1.172	2.165
Actinomyces dentalis	Common	1.364	2.197	*Lautropia mirabilis*	Common	1.156	0.526
Capnocytophaga leadbetteri	Common	1.174	1.512	*Neisseria elongata*	Common	1.069	1.500
Leptotrichia shahii	Common	1.012	1.823	*Corynebacterium durum*	Common	1.049	1.097
Moraxella catarrhalis	Common	0.973	1.917	*Abiotrophia defectiva*	Common	1.048	0.452
Selenomonas sputigena	Common	0.954	0.846	*Streptococcus cristatus*	Common	1.034	2.068
Veillonella dispar	Common	0.851	0.558	*Neisseria mucosa*	Common	1.025	1.509
Kingella denitrificans	Common	0.833	1.882	*Capnocytophaga leadbetteri*	Common	0.948	1.270
Haemophilus parainfluenzae	Common	0.751	1.232	*Actinomyces oris*	Common	0.897	0.864
Actinomyces oris	Common	0.749	1.790	*Rothia dentocariosa*	Common	0.839	1.153
Campylobacter gracilis	Common	0.710	0.638	*Capnocytophaga sputigena*	Common	0.777	0.829
Streptococcus dentisani	Common	0.700	1.022	*Actinomyces dentalis*	Common	0.747	1.169
Corynebacterium durum	Common	0.617	0.755	*Actinomyces massiliensis*	Common	0.682	0.642

Rothia aeria	Common	0.614	0.810	*Veillonella dispar*	Common	0.646	0.549
Neisseria elongata	Common	0.589	0.667	*Capnocytophaga gingivalis*	Common	0.613	0.691
Neisseria subflava	Common	0.548	1.195	*Leptotrichia shahii*	Common	0.599	1.198
Cardiobacterium valvarum	Common	0.533	0.747	*Granulicatella adiacens*	Common	0.507	0.548
Actinomyces johnsonii	Common	0.523	0.788	*Streptococcus sanguinis*	Common	0.453	0.523
Actinomyces georgiae	Specific	0.515	1.262	*Leptotrichia hofstadii*	Common	0.452	0.709
Lachnoanaerobaculum umeaense	Common	0.467	0.726	*Fusobacterium periodonticum*	Common	0.429	0.754
Rothia dentocariosa	Common	0.455	0.571	*Porphyromonas catoniae*	Common	0.422	0.618
Lachnoanaerobaculum saburreum	Common	0.455	1.114	*Neisseria pharyngis*	Specific	0.418	0.833
Abiotrophia defectiva	Common	0.445	1.014	*Kingella denitrificans*	Common	0.415	0.206
Capnocytophaga ochracea	Specific	0.443	1.085	*Gemella haemolysans*	Common	0.376	0.510
Capnocytophaga sputigena	Common	0.412	0.531	*Streptococcus lactarius*	Common	0.366	0.732
Porphyromonas catoniae	Common	0.345	0.609	*Actinomyces odontolyticus*	Common	0.346	0.419
Kingella oralis	Common	0.342	0.317	*Veillonella parvula*	Common	0.343	0.625
Leptotrichia hongkongensis	Common	0.326	0.698	*Ottowia* sp.	Common	0.334	0.465
Streptococcus mutans	Common	0.297	0.720	*Gemella morbillorum*	Common	0.304	0.272
Actinomyces gerencseriae	Common	0.296	0.402	*Streptococcus gordonii*	Common	0.304	0.608
Granulicatella adiacens	Common	0.289	0.277	*Leptotrichia wadei*	Common	0.287	0.575
Neisseria polysaccharea	Specific	0.284	0.695	*Enterococcus italicus*	Specific	0.286	0.320
Actinomyces viscosus	Specific	0.247	0.604	*Veillonella atypica*	Common	0.283	0.433
Fusobacterium naviforme	Specific	0.235	0.575	*Propionibacterium propionicum*	Common	0.263	0.143
Streptococcus lactarius	Common	0.184	0.451	*Rothia aeria*	Common	0.236	0.168
Prevotella oulorum	Common	0.182	0.191	*Leptotrichia hongkongensis*	Common	0.227	0.187
Gemella morbillorum	Common	0.181	0.109	*Prevotella melaninogenica*	Common	0.225	0.234
Selenomonas flueggei	Specific	0.165	0.298	*Neisseria oralis*	Common	0.211	0.250
Stomatobaculum longum	Common	0.161	0.262	*Selenomonas sputigena*	Common	0.166	0.190
Propionibacterium propionicum	Common	0.154	0.107	*Gemella sanguinis*	Common	0.144	0.288
Selenomonas noxia	Common	0.144	0.312	*Centipeda periodontii*	Common	0.142	0.284
Gemella sanguinis	Common	0.120	0.186	*Streptococcus anginosus*	Common	0.140	0.211
Prevotella nigrescens	Common	0.112	0.109	*Actinomyces johnsonii*	Common	0.139	0.259
Streptococcus mitis	Common	0.111	0.235	*Cardiobacterium valvarum*	Common	0.131	0.249

Neisseria flava	Specific	0.104	0.166	*Campylobacter concisus*	Common	0.129	0.117
Veillonella atypica	Common	0.103	0.208	*Selenomonas infelix*	Common	0.118	0.236
Streptococcus oligofermentans	Common	0.099	0.243	*Campylobacter gracilis*	Common	0.117	0.234
Lactobacillus vaginalis	Specific	0.095	0.233	*Kingella oralis*	Common	0.102	0.071
Selenomonas infelix	Common	0.087	0.121	*Prevotella nigrescens*	Common	0.097	0.104
Capnocytophaga gingivalis	Common	0.081	0.121	*Lachnoanaerobaculum saburreum*	Common	0.093	0.187
Neisseria flavescens	Common	0.079	0.140	*Aggregatibacter paraphrophilus*	Common	0.083	0.166
Campylobacter concisus	Common	0.077	0.096	*Catonella morbi*	Common	0.082	0.107
Prevotella scopos	Specific	0.073	0.180	*Actinomyces gerencseriae*	Common	0.078	0.103
Fusobacterium periodonticum	Common	0.073	0.136	*Selenomonas noxia*	Common	0.071	0.102
Atopobium parvulum	Specific	0.072	0.174	*Streptococcus parasanguinis*	Common	0.068	0.136
Veillonella parvula	Common	0.071	0.084	*Eikenella corrodens*	Common	0.065	0.130
Actinomyces odontolyticus	Common	0.069	0.110	*Selenomonas artemidis*	Common	0.057	0.093
Streptococcus tigurinus	Specific	0.064	0.125	*Actinomyces israelii*	Specific	0.043	0.086
Actinomyces timonensis	Specific	0.061	0.106	*Mitsuokella multacida*	Specific	0.031	0.062
Prevotella melaninogenica	Common	0.060	0.043	*Prevotella oulorum*	Common	0.030	0.027
Granulicatella elegans	Common	0.057	0.141	*Porphyromonas endodontalis*	Specific	0.030	0.061
Aggregatibacter segnis	Common	0.056	0.137	*Megasphaera micronuciformis*	Common	0.030	0.060
Streptococcus anginosus	Common	0.044	0.107	*Prevotella pallens*	Common	0.029	0.038
Gemella haemolysans	Common	0.040	0.055	*Aggregatibacter segnis*	Common	0.027	0.055
Catonella morbi	Common	0.040	0.038	*Granulicatella elegans*	Common	0.026	0.019
Centipeda periodontii	Common	0.038	0.042	*Prevotella saccharolytica*	Common	0.026	0.029
Prevotella maculosa	Common	0.038	0.055	*Streptococcus oligofermentans*	Common	0.024	0.048
Selenomonas artemidis	Common	0.034	0.048	*Johnsonella ignava*	Common	0.023	0.046
Dialister invisus	Common	0.034	0.069	*Stomatobaculum longum*	Common	0.020	0.036
Prevotella micans	Common	0.034	0.042	*Streptococcus intermedius*	Common	0.017	0.022
Johnsonella ignava	Common	0.033	0.079	*Solobacterium moorei*	Common	0.014	0.016
Prevotella veroralis	Common	0.033	0.071	*Dialister invisus*	Common	0.012	0.014
Prevotella saccharolytica	Common	0.028	0.018	*Prevotella denticola*	Common	0.011	0.019
Prevotella salivae	Common	0.028	0.041	*Veillonella denticariosi*	Common	0.011	0.023
Leptotrichia goodfellowii	Common	0.028	0.039	*Streptococcus mutans*	Common	0.010	0.011

Streptococcus cristatus	Common	0.025	0.061
Streptococcus parasanguinis	Common	0.022	0.055
Streptococcus gordonii	Common	0.019	0.048
Prevotella denticola	Common	0.019	0.037
Prevotella intermedia	Specific	0.018	0.045
Prevotella oris	Common	0.016	0.019
Eikenella corrodens	Common	0.016	0.038
Streptococcus intermedius	Common	0.016	0.025
Megasphaera micronuciformis	Common	0.015	0.023
Alloprevotella tannerae	Specific	0.014	0.014
Lactobacillus fermentum	Specific	0.013	0.033
Aggregatibacter paraphrophilus	Common	0.013	0.033
Rothia mucilaginosa	Specific	0.013	0.032
Selenomonas dianae	Specific	0.012	0.031
Atopobium rimae	Common	0.011	0.023
Streptococcus salivarius	Specific	0.011	0.026
Prevotella nanceiensis	Common	0.008	0.013
Aggregatibacter aphrophilus	Specific	0.008	0.019
Oribacterium sinus	Common	0.007	0.017
Ottowia sp.	Common	0.006	0.015
Treponema socranskii	Common	0.004	0.005
Sneathia amnii	Specific	0.004	0.009
Solobacterium moorei	Common	0.004	0.006
Scardovia wiggsiae	Specific	0.003	0.008
Prevotella marshii	Specific	0.003	0.007
Bifidobacterium dentium	Specific	0.003	0.008
Mogibacterium diversum	Common	0.003	0.008
Oribacterium asaccharolyticum	Specific	0.003	0.007
Shuttleworthia satelles	Specific	0.002	0.006
Parvimonas micra	Common	0.002	0.006
Haemophilus haemolyticus	Common	0.002	0.006
Prevotella maculosa	Common	0.010	0.008
Streptococcus dentisani	Common	0.009	0.018
Treponema socranskii	Common	0.009	0.011
Oribacterium parvum	Specific	0.008	0.017
Capnocytophaga haemolytica	Specific	0.008	0.007
Prevotella salivae	Common	0.008	0.017
Haemophilus parahaemolyticus	Specific	0.008	0.016
Prevotella micans	Common	0.007	0.009
Prevotella oris	Common	0.007	0.009
Oribacterium sinus	Common	0.006	0.009
Mogibacterium diversum	Common	0.005	0.006
Alloprevotella rava	Specific	0.004	0.005
Parvimonas micra	Common	0.003	0.007
Prevotella loescheii	Specific	0.003	0.006
Erysipelothrix tonsillarum	Specific	0.003	0.005
Haemophilus paraphrohaemolyticus	Specific	0.002	0.004
Haemophilus haemolyticus	Common	0.002	0.004
Leptotrichia goodfellowii	Common	0.002	0.004
Atopobium rimae	Common	0.002	0.003
Enterococcus durans	Common	0.002	0.003
Prevotella veroralis	Common	0.001	0.003
Prevotella nanceiensis	Common	0.001	0.003
Unclassified sp.	Common	25.76	7.045

Prevotella pallens	Common	0.002	0.004
Lactobacillus rhamnosus	Specific	0.002	0.005
Anaeroglobus geminatus	Specific	0.002	0.004
Veillonella denticariosi	Common	0.002	0.005
Mycoplasma salivarium	Specific	0.001	0.002
Prevotella pleuritidis	Specific	0.001	0.002
Enterococcus durans	Common	0.001	0.001
Unclassified sp.	Common	29.36	4.937

Table S3. Rank distribution of the level-1 SEED subsystem functions assigned for the mRNA reads (relative abundance per participant).

CLP			Control		
Function	Mean	Standard deviation	Function	Mean	Standard deviation
Protein Metabolism	24.727	2.963	Carbohydrates	21.035	6.015
Carbohydrates	15.629	1.305	Protein Metabolism	16.721	2.711
Clustering-based subsystems	11.805	0.664	Clustering-based subsystems	11.466	1.954
Amino Acids and Derivatives	6.951	0.754	Amino Acids and Derivatives	7.344	0.721
Miscellaneous	4.543	0.745	Cofactors, Vitamins, Prosthetic Groups, Pigments	4.620	0.501
Cofactors, Vitamins, Prosthetic Groups, Pigments	4.440	0.303	Respiration	4.346	0.944
RNA Metabolism	4.177	0.209	Miscellaneous	4.182	1.328
Membrane Transport	3.731	1.183	RNA Metabolism	3.960	1.078
Nucleosides and Nucleotides	3.399	0.364	Membrane Transport	3.830	0.634
Respiration	3.253	0.507	Cell Wall and Capsule	3.802	0.795
DNA Metabolism	3.229	0.868	Nucleosides and Nucleotides	3.493	0.783
Stress Response	2.828	0.459	DNA Metabolism	3.249	1.297
Cell Wall and Capsule	2.547	0.225	Stress Response	2.253	0.435
Fatty Acids, Lipids, and Isoprenoids	1.459	0.311	Virulence, Disease and Defense	1.668	0.374
Virulence, Disease and Defense	1.433	0.145	Fatty Acids, Lipids, and Isoprenoids	1.605	0.268
Phages, Prophages, Transposable elements, Plasmids	0.983	0.292	Iron acquisition and metabolism	1.194	0.795
Nitrogen Metabolism	0.863	0.378	Cell Division and Cell Cycle	0.978	0.343
Cell Division and Cell Cycle	0.804	0.148	Nitrogen Metabolism	0.937	0.572
Iron acquisition and metabolism	0.800	0.224	Phages, Prophages, Transposable elements, Plasmids	0.756	0.123
Phosphorus Metabolism	0.515	0.132	Regulation and Cell signaling	0.602	0.149
Sulfur Metabolism	0.503	0.134	Sulfur Metabolism	0.466	0.204
Regulation and Cell signaling	0.495	0.175	Phosphorus Metabolism	0.459	0.220
Motility and Chemotaxis	0.283	0.072	Motility and Chemotaxis	0.381	0.191
Metabolism of Aromatic Compounds	0.278	0.155	Metabolism of Aromatic Compounds	0.324	0.104
Potassium Metabolism	0.130	0.100	Potassium Metabolism	0.135	0.068
Secondary Metabolism	0.097	0.044	Secondary Metabolism	0.106	0.076

Dormancy and Sporulation	0.076	0.034	Dormancy and Sporulation	0.075	0.057
Photosynthesis	0.023	0.035	Photosynthesis	0.016	0.020

Table S4. Rank distribution of the level-2 KEGG functions assigned for the mRNA reads (relative abundance per participant).

CLP				Control			
Function	Group-specificity	Mean	Standard deviation	Function	Group-specificity	Mean	Standard deviation
Translation	Common	20.45	2.903	Carbohydrate metabolism	Common	16.41	2.439
Amino acid metabolism	Common	14.09	1.218	Amino acid metabolism	Common	15.29	0.817
Carbohydrate metabolism	Common	13.24	0.669	Translation	Common	15.16	2.272
Membrane transport	Common	9.654	0.805	Membrane transport	Common	10.02	1.382
Signal transduction	Common	5.490	0.661	Signal transduction	Common	5.862	1.180
Metabolism of cofactors and vitamins	Common	4.506	0.513	Energy metabolism	Common	5.034	0.704
Energy metabolism	Common	4.482	0.562	Metabolism of cofactors and vitamins	Common	4.932	1.382
Nucleotide metabolism	Common	4.471	0.726	Nucleotide metabolism	Common	4.278	0.654
Replication and repair	Common	4.168	0.974	Replication and repair	Common	4.104	1.320
Folding	Common	3.075	0.680	Lipid metabolism	Common	2.946	0.510
Environmental Adaptation	Common	3.035	0.788	Folding	Common	2.578	0.349
Transcription	Common	2.900	0.225	Transcription	Common	2.446	0.183
Lipid metabolism	Common	2.359	0.238	Glycan biosynthesis and metabolism	Common	1.850	0.562
Cell growth and death	Common	1.589	0.441	Environmental Adaptation	Common	1.782	0.546
Glycan biosynthesis and metabolism	Common	1.525	0.163	Cell growth and death	Common	1.332	0.339
Metabolism of terpenoids and polyketides	Common	0.859	0.211	Metabolism of terpenoids and polyketides	Common	0.919	0.209
Transport and catabolism	Common	0.795	0.193	Biosynthesis of Other Secondary Metabolites	Common	0.892	0.198
Biosynthesis of Other Secondary Metabolites	Common	0.717	0.138	Transport and catabolism	Common	0.868	0.195
Metabolism of other amino acids	Common	0.692	0.053	Infectious diseases	Common	0.863	0.120
Cancers	Common	0.571	0.164	Metabolism of other amino acids	Common	0.862	0.271
Cell motility	Common	0.534	0.104	Cell motility	Common	0.672	0.300
Infectious diseases	Common	0.533	0.078	Cancers	Common	0.527	0.253
Xenobiotics biodegradation and metabolism	Common	0.104	0.055	Xenobiotics biodegradation and metabolism	Common	0.162	0.035

Immune diseases	Common	0.063	0.067	Digestive system	Common	0.078	0.053
Digestive system	Common	0.041	0.031	Immune diseases	Common	0.054	0.061
Neurodegenerative diseases	Specific	0.014	0.020	Endocrine system	Common	0.054	0.032
Endocrine system	Common	0.014	0.013	Immune system	Specific	0.010	0.021
Cell communication	Common	0.005	0.011	Cell communication	Common	0.007	0.008
Substance dependence	Specific	0.002	0.006				
Development	Specific	0.002	0.005				

Table S5. Rank distribution of the top 50 species assigned for the mRNA reads (relative abundance per participant).

CLP			Control		
Species	Mean	Standard deviation	Species	Mean	Standard deviation
Corynebacterium matruchotii	4.906	3.735	*Leptotrichia hofstadii*	4.089	4.282
Leptotrichia hofstadii	4.582	2.971	*Corynebacterium matruchotii*	3.150	2.663
Actinomyces sp.	3.421	2.128	*Actinomyces* sp.	2.873	1.656
Fusobacterium nucleatum	2.872	1.440	*Actinomyces naeslundii*	2.576	1.015
Actinomyces naeslundii	2.838	1.082	*Streptococcus pneumoniae*	2.549	0.901
Capnocytophaga sp.	2.385	1.341	*Fusobacterium nucleatum*	2.548	1.852
Actinomyces johnsonii	2.151	2.634	*Capnocytophaga* sp.	2.475	1.286
Tannerella sp.	2.147	1.906	*Streptococcus* sp.	2.307	2.569
Leptotrichia sp.	1.965	0.338	*Neisseria* sp.	2.169	2.477
Prevotella sp.	1.831	0.996	*Actinobaculum* sp.	1.865	3.130
Mycobacterium tuberculosis	1.819	1.656	*Streptococcus oralis*	1.731	2.233
Streptococcus pneumoniae	1.770	1.011	*Mycobacterium tuberculosis*	1.713	1.918
Selenomonas sp.	1.369	0.746	*Actinomyces oris*	1.480	0.870
Actinomyces oris	1.320	0.632	*Leptotrichia* sp.	1.457	0.555
Actinomyces massiliensis	1.226	0.707	*Streptococcus sanguinis*	1.344	0.036
Unclassified sp.	1.106	0.803	*Prevotella* sp.	1.232	0.850
Rhodococcus sp.	1.082	0.979	*Veillonella* sp.	1.137	1.036
Lachnoanaerobaculum saburreum	1.031	0.778	*Porphyromonas* sp.	1.109	1.070
Streptomyces sp.	1.016	1.057	Unclassified sp.	1.071	1.005
Corynebacterium pseudogenitalium	1.011	1.264	*Tannerella* sp.	1.070	1.340
Selenomonas noxia	0.992	0.786	*Neisseria meningitidis*	1.052	0.614
Bifidobacterium adolescentis	0.964	0.869	*Rhodococcus* sp.	0.991	1.150
Ruminococcus torques	0.902	0.829	*Veillonella parvula*	0.965	0.977
Propionibacterium acnes	0.877	1.012	*Streptomyces* sp.	0.897	1.092
Staphylococcus aureus	0.871	0.794	*Corynebacterium durum*	0.877	0.836
Corynebacterium efficiens	0.854	0.893	*Actinomyces massiliensis*	0.845	0.633
Streptococcus sp.	0.851	0.321	*Ruminococcus torques*	0.838	0.970

Neisseria sp.	0.836	0.582	*Corynebacterium pseudogenitalium*	0.787	1.014
Actinomyces dentalis	0.779	0.648	*Bifidobacterium adolescentis*	0.774	0.900
Leptotrichia wadei	0.752	0.619	*Haemophilusparainfluenzae*	0.772	0.830
Veillonella sp.	0.722	0.097	*Staphylococcus aureus*	0.710	0.860
Pseudopropionibacterium propionicum	0.698	0.662	*Corynebacterium efficiens*	0.690	0.815
Streptococcus sanguinis	0.685	0.532	*Veillonella dispar*	0.679	0.620
Daphnia magna	0.669	0.610	*Actinomyces odontolyticus*	0.672	0.498
Neisseria meningitidis	0.634	0.121	*Cardiobacterium hominis*	0.671	0.644
Olsenella sp.	0.634	0.583	*Streptococcus gordonii*	0.661	0.881
Veillonella parvula	0.613	0.251	*Propionibacterium acnes*	0.617	0.711
Streptococcus oralis	0.602	0.268	*Porphyromonas catoniae*	0.612	0.881
Porphyromonas sp.	0.558	0.263	*Capnocytophaga gingivalis*	0.603	0.347
Campylobacter showae	0.554	0.566	*Neisseria sicca*	0.573	0.878
Ruminococcus gnavus	0.549	0.505	*Pseudopropionibacterium propionicum*	0.566	0.547
Actinomyces odontolyticus	0.537	0.227	*Daphnia magna*	0.564	0.685
Nocardia asteroides	0.511	0.725	*Streptococcus mitis*	0.517	0.577
Campylobacter jejuni	0.448	0.374	*Selenomonas* sp.	0.497	0.283
Cardiobacterium hominis	0.422	0.210	*Ruminococcus gnavus*	0.490	0.565
Capnocytophaga ochracea	0.410	0.384	*Abiotrophia* sp.	0.487	0.568
Actinomyces turicensis	0.402	0.419	*Neisseria elongata*	0.481	0.457
Blautia obeum	0.401	0.367	*Lautropia mirabilis*	0.471	0.373
Veillonella dispar	0.400	0.088	*Capnocytophaga granulosa*	0.464	0.289
Fusobacterium sp.	0.400	0.269	*Abiotrophia defectiva*	0.462	0.437

Table S6. Rank distribution of VTiF (mean RPKM values among the participants and the mRNA-to-rRNA ratio).

CLP				Control			
Species	rc-rRNA	mRNA	log2 mRNA-to-rRNA ratio	Species	rc-rRNA	mRNA	log2 mRNA-to-rRNA ratio
Enterococcus durans	1.2	2132.6	10.81	*Haemophilus haemolyticus*	4.2	8079.4	10.91
Lactobacillus rhamnosus	3.9	6519.9	10.70	*Prevotella loescheii*	6.6	6497.1	9.95
Haemophilus haemolyticus	6.0	7702.1	10.31	*Enterococcus durans*	3.4	2421.7	9.49
Lactobacillus fermentum	26.5	16924.4	9.32	*Leptotrichia goodfellowii*	4.1	2795.8	9.43
Treponema socranskii	8.8	4998.8	9.15	*Leptotrichia hofstadii*	1168.4	411882	8.46
Streptococcus salivarius	27.2	13784.4	8.99	*Streptococcus intermedius*	37.5	10647.1	8.15
Prevotella pallens	4.8	2126.7	8.79	*Oribacterium sinus*	15.2	3886.9	8.00
Aggregatibacter aphrophilus	14.3	5883.0	8.69	*Stomatobaculum longum*	53.7	13088.0	7.93
Actinomyces odontolyticus	118.3	43568.5	8.52	*Prevotella veroralis*	3.1	668.1	7.75
Veillonella parvula	116.9	40951.6	8.45	*Prevotella oris*	18.0	2233.0	6.96
Streptococcus gordonii	50.1	16859.6	8.40	*Parvimonas micra*	7.4	873.5	6.88
Streptococcus intermedius	37.9	11772.5	8.28	*Lachnoanaerobaculum saburreum*	210.2	23158.6	6.78
Streptococcus cristatus	47.3	13332.4	8.14	*Selenomonas noxia*	182.1	18586.5	6.67
Eikenella corrodens	30.7	8047.6	8.03	*Dialister invisus*	30.8	2989.9	6.60
Solobacterium moorei	7.7	1943.7	7.97	*Prevotella nigrescens*	246.5	18887.2	6.26
Centipeda periodontii	81.2	20222.8	7.96	*Treponema socranskii*	23.2	1771.8	6.25
Oribacterium sinus	15.6	3712.6	7.89	*Streptococcus sanguinis*	1055.0	76975.6	6.19
Prevotella pleuritidis	2.0	400.6	7.64	*Prevotella maculosa*	23.9	1571.7	6.04
Leptotrichia hofstadii	2453.6	422558	7.43	*Kingella oralis*	236.3	13156.7	5.80
Dialister invisus	43.7	6535.9	7.23	*Prevotella saccharolytica*	62.9	3488.8	5.79
Prevotella saccharolytica	52.8	7956.2	7.23	*Prevotella salivae*	19.9	1080.8	5.76
Capnocytophaga gingivalis	151.9	21037.5	7.11	*Actinomyces odontolyticus*	759.6	40572.8	5.74
Parvimonas micra	6.2	804.8	7.02	*Atopobium rimae*	4.5	239.2	5.73
Selenomonas noxia	320.4	41595.9	7.02	*Actinomyces gerencseriae*	198.1	10405.9	5.72
Prevotella marshii	6.6	782.7	6.88	*Veillonella parvula*	717.1	34755.7	5.60
Prevotella nigrescens	205.2	23879.6	6.86	*Johnsonella ignava*	55.9	2450.2	5.46
Leptotrichia goodfellowii	44.8	5080.1	6.83	*Solobacterium moorei*	32.8	1436.8	5.45

Prevotella maculosa	56.8	5775.0	6.67	*Centipeda periodontii*	378.1	16272.0	5.43
Prevotella oris	37.6	3511.1	6.55	*Streptococcus mutans*	21.6	932.0	5.43
Prevotella veroralis	38.8	3637.0	6.55	*Fusobacterium nucleatum*	5421.8	222092	5.36
Prevotella micans	51.0	4524.7	6.47	*Campylobacter gracilis*	310.7	12356.7	5.31
Streptococcus anginosus	85.6	7521.2	6.46	*Capnocytophaga haemolytica*	19.0	717.9	5.24
Ottowia sp.	11.8	983.7	6.38	*Eikenella corrodens*	156.3	5694.5	5.19
Actinomyces johnsonii	773.8	64192.7	6.37	*Actinomyces johnsonii*	331.1	11541.7	5.12
Catonella morbi	78.2	6157.2	6.30	*Actinomyces israelii*	88.6	2909.1	5.04
Selenomonas artemidis	81.7	5375.9	6.04	*Streptococcus gordonii*	629.1	19427.8	4.95
Anaeroglobus geminatus	3.9	240.4	5.93	*Prevotella denticola*	27.5	842.9	4.94
Johnsonella ignava	64.8	3678.3	5.83	*Prevotella oulorum*	75.7	2279.2	4.91
Prevotella melaninogenica	136.9	7658.6	5.81	*Rothia aeria*	564.1	15569.3	4.79
Lachnoanaerobaculum saburreum	1014.4	54952.1	5.76	*Actinomyces massiliensis*	1610.4	41295.6	4.68
Streptococcus mitis	288.3	15237.8	5.72	*Prevotella nanceiensis*	2.9	73.8	4.68
Prevotella intermedia	49.6	2587.9	5.71	*Actinomyces oris*	2174.8	53884.4	4.63
Fusobacterium nucleatum	4637.5	241619	5.70	*Prevotella pallens*	72.2	1676.9	4.54
Neisseria flavescens	155.8	7783.3	5.64	*Haemophilus paraphrohaemolyticus*	4.6	96.0	4.38
Actinomyces oris	1665.9	82469.1	5.63	*Aggregatibacter segnis*	56.8	1174.0	4.37
Oribacterium asaccharolyticum	6.0	293.5	5.62	*Actinomyces naeslundii*	5360.9	105885	4.30
Prevotella denticola	39.0	1920.8	5.62	*Selenomonas artemidis*	134.2	2617.4	4.29
Fusobacterium periodonticum	138.6	5959.1	5.43	*Oribacterium parvum*	19.0	355.9	4.22
Campylobacter concisus	180.6	7560.7	5.39	*Streptococcus anginosus*	364.8	6741.9	4.21
Selenomonas flueggei	232.2	9499.4	5.35	*Catonella morbi*	200.3	3649.1	4.19
Alloprevotella tannerae	30.0	1154.7	5.27	*Prevotella micans*	18.8	340.4	4.18
Prevotella salivae	59.6	2295.2	5.27	*Leptotrichia wadei*	763.8	12120.2	3.99
Prevotella oulorum	307.3	11711.7	5.25	*Capnocytophaga gingivalis*	1406.4	22049.4	3.97
Selenomonas infelix	173.4	6561.8	5.24	*Veillonella dispar*	1520.4	23347.1	3.94
Actinomyces naeslundii	4401.6	148917	5.08	*Campylobacter concisus*	321.7	4581.7	3.83
Aggregatibacter segnis	124.7	3796.8	4.93	*Selenomonas infelix*	283.3	3864.6	3.77

Stomatobaculum longum	352.4	10558.8	4.91
Gemella morbillorum	344.9	10231.3	4.89
Actinomyces viscosus	634.8	18355.2	4.85
Gemella haemolysans	102.6	2937.4	4.84
Scardovia wiggsiae	6.7	170.2	4.66
Bifidobacterium dentium	6.4	154.0	4.58
Actinomyces gerencseriae	664.3	15556.3	4.55
Megasphaera micronuciformis	34.1	721.0	4.40
Actinomyces massiliensis	3586.1	67149.2	4.23
Shuttleworthia satelles	4.9	91.6	4.23
Prevotella scopos	163.6	2894.6	4.14
Atopobium rimae	22.0	352.1	4.00
Rothia mucilaginosa	33.7	511.3	3.92
Capnocytophaga ochracea	1140.2	16417.5	3.85
Capnocytophaga sputigena	1040.6	14754.8	3.83
Actinomyces dentalis	2544.0	35162.7	3.79
Veillonella dispar	1820.8	25202.7	3.79
Campylobacter gracilis	1440.2	19572.0	3.76
Kingella oralis	820.0	10869.2	3.73
Corynebacterium durum	1436.8	17903.9	3.64
Haemophilus parainfluenzae	1909.7	23518.0	3.62
Porphyromonas catoniae	853.7	10248.8	3.59
Corynebacterium matruchotii	18244.8	219299	3.59
Rothia aeria	1257.9	15017.9	3.58
Streptococcus sanguinis	7271.1	75543.2	3.38
Streptococcus mutans	585.2	5496.8	3.23
Cardiobacterium valvarum	1003.3	9146.8	3.19
Veillonella atypica	254.7	2306.6	3.18
Actinomyces timonensis	130.5	991.3	2.93
Neisseria elongata	1413.3	10485.1	2.89
Abiotrophia defectiva	1185.4	8756.8	2.88

Alloprevotella rava	10.6	144.3	3.76
Granulicatella elegans	62.4	831.3	3.74
Selenomonas sputigena	415.5	4840.3	3.54
Cardiobacterium valvarum	344.8	3951.6	3.52
Prevotella melaninogenica	567.0	6078.6	3.42
Porphyromonas catoniae	1000.7	10529.6	3.40
Streptococcus oralis	4861.0	50371.1	3.37
Corynebacterium matruchotii	12768.0	121702	3.25
Gemella morbillorum	705.2	5936.1	3.07
Corynebacterium durum	2276.9	16501.9	2.86
Capnocytophaga sputigena	1692.4	10960.1	2.70
Actinomyces dentalis	1949.8	12381.7	2.67
Abiotrophia defectiva	2462.5	11744.0	2.25
Kingella denitrificans	939.6	4392.8	2.23
Lautropia mirabilis	2687.5	12154.8	2.18
Streptococcus mitis	3152.0	13847.4	2.14
Haemophilus parahaemolyticus	18.9	77.6	2.04
Neisseria elongata	2518.4	10161.4	2.01
Granulicatella adiacens	1116.5	4368.2	1.97
Haemophilus parainfluenzae	6872.5	25308.2	1.88
Rothia dentocariosa	1796.0	6616.0	1.88
Fusobacterium periodonticum	1041.0	3666.2	1.82
Gemella haemolysans	835.6	2825.6	1.76
Streptococcus cristatus	2749.1	9147.2	1.73
Mitsuokella multacida	82.8	239.9	1.54
Gemella sanguinis	346.2	960.9	1.47
Megasphaera micronuciformis	79.8	203.0	1.35
Ottowia sp.	765.1	1809.0	1.24
Capnocytophaga granulosa	11111.9	24260.7	1.13
Neisseria mucosa	2634.5	5564.8	1.08
Leptotrichia shahii	1439.5	2773.5	0.95

Prevotella nanceiensis	19.9	132.9	2.74
Granulicatella adiacens	642.8	4248.6	2.72
Campylobacter showae	3175.0	20021.6	2.66
Selenomonas sputigena	1697.2	10022.3	2.56
Gemella sanguinis	248.2	1384.1	2.48
Mycoplasma salivarium	2.4	12.3	2.38
Rothia dentocariosa	1010.7	5202.1	2.36
Granulicatella elegans	154.5	728.9	2.24
Cardiobacterium hominis	6377.1	28956.1	2.18
Actinomyces georgiae	1148.8	5178.4	2.17
Leptotrichia shahii	1768.2	7331.0	2.05
Atopobium parvulum	141.3	543.5	1.94
Leptotrichia wadei	10393.8	32457.5	1.64
Capnocytophaga granulosa	11270.5	32781.1	1.54
Lautropia mirabilis	3884.1	10912.6	1.49
Neisseria subflava	671.4	1557.1	1.21
Neisseria sicca	4789.3	10371.3	1.11
Kingella denitrificans	2221.4	4539.1	1.03
Leptotrichia buccalis	7514.9	13263.2	0.82
Neisseria polysaccharea	729.7	862.6	0.24
Lactobacillus vaginalis	186.5	170.2	-0.13
Neisseria mucosa	4624.7	3996.8	-0.21
Moraxella catarrhalis	2478.3	1393.9	-0.83
Leptotrichia hongkongensis	841.2	132.4	-2.67
Neisseria flava	216.7	31.7	-2.77
Streptococcus lactarius	474.3	64.9	-2.87
Neisseria oralis	5009.2	13.6	-8.52

Veillonella atypica	739.1	1364.9	0.88
Campylobacter showae	2800.8	4908.9	0.81
Cardiobacterium hominis	12059.3	20247.5	0.75
Porphyromonas endodontalis	73.0	69.5	-0.07
Neisseria flavescens	7378.1	6103.7	-0.27
Neisseria sicca	21237.3	9226.0	-1.20
Neisseria subflava	2752.1	1170.1	-1.23
Leptotrichia buccalis	9911.7	3342.4	-1.57
Moraxella catarrhalis	6386.8	1206.6	-2.40
Streptococcus lactarius	879.2	79.2	-3.47
Leptotrichia hongkongensis	523.8	41.3	-3.67
Veillonella denticariosi	23.3	1.3	-4.16
Neisseria oralis	528.6	11.0	-5.59
Neisseria pharyngis	1004.6	18.4	-5.77

Table S7. Rank distribution of the top 50 VFDB functions assigned for the mRNA reads (relative abundance per participant).

CLP				Control			
Function	Group-specificity	Mean	Standard deviation	Function	Group-specificity	Mean	Standard deviation
elongation factor Tu	Common	4.988	1.235	elongation factor Tu	Common	3.429	1.171
translation elongation factor Tu	Common	4.049	1.567	glyceraldehyde-3-phosphate dehydrogenase	Common	3.175	1.534
surface-anchored fimbrial subunit	Common	3.741	3.033	surface-anchored fimbrial subunit	Common	3.002	2.324
glyceraldehyde-3-phosphate dehydrogenase	Common	3.352	0.924	hypothetical protein	Common	2.944	0.790
hypothetical protein	Common	2.890	0.421	translation elongation factor Tu	Common	2.840	1.115
glyceraldehyde-3-phosphate dehydrogenase, type I	Common	2.535	0.772	phosphopyruvate hydratase	Common	2.797	1.776
phosphopyruvate hydratase	Common	2.333	0.819	glyceraldehyde-3-phosphate dehydrogenase, type I	Common	2.286	1.342
Unspecified	Common	1.656	0.411	Unspecified	Common	1.886	0.480
Elongation factor Tu	Common	1.609	0.660	type IV pilin structural subunit	Common	1.714	2.980
FimP	Common	1.428	2.022	molecular chaperone DnaK	Common	1.121	0.310
chaperonin GroEL	Common	1.311	0.816	ATP-dependent Clp protease proteolytic subunit	Common	1.113	0.233
ATP-dependent Clp protease proteolytic subunit	Common	1.260	0.304	Elongation factor Tu	Common	0.988	0.303
molecular chaperone DnaK	Common	1.259	0.732	FimP	Common	0.868	0.564
endopeptidase Clp ATP-binding chain C	Common	0.862	0.336	chaperonin GroEL	Common	0.819	0.284
Glyceraldehyde 3-phosphate dehydrogenase, putative	Common	0.775	0.587	ABC transporter ATP-binding protein	Common	0.811	0.235
ABC transporter ATP-binding protein	Common	0.718	0.177	enolase	Common	0.790	0.457
enolase	Common	0.685	0.213	endopeptidase Clp ATP-binding chain C	Common	0.696	0.122
3-oxoacyl-ACP reductase	Common	0.664	0.168	acyl carrier protein	Common	0.586	0.229

RNA polymerase sigma factor	Common	0.572	0.124	Glyceraldehyde 3-phosphate dehydrogenase, putative	Common	0.581	0.275
acyl carrier protein	Common	0.538	0.239	3-oxoacyl-ACP reductase	Common	0.579	0.111
ABC transporter ATP-binding protein AatC	Common	0.530	0.241	UDP-glucose 4-epimerase	Common	0.541	0.265
oligopeptide ABC transporter, permease component	Common	0.492	0.149	phaseolotoxin-insensitive ornithine carbamoyltransferase	Common	0.509	0.156
UDP-glucose 4-epimerase	Common	0.440	0.113	SpaH-type pili major subunit SpaH	Common	0.492	0.797
two-component system response regulator	Common	0.438	0.245	oligopeptide ABC transporter, permease component	Common	0.483	0.133
ABC transporter, ATP-binding protein	Common	0.403	0.182	ABC transporter, ATP-binding protein	Common	0.481	0.176
serine/threonine protein kinase	Common	0.396	0.086	superoxide dismutase	Common	0.475	0.114
glutamine synthetase	Common	0.389	0.143	ABC transporter ATP-binding protein AatC	Common	0.436	0.102
superoxide dismutase	Common	0.387	0.155	Dot/Icm type IV secretion system effector LirB	Common	0.432	0.153
immunogenic lipoprotein A	Common	0.374	0.156	Enolase, putative	Common	0.430	0.216
Enolase, putative	Common	0.371	0.111	flagellin subunit	Common	0.428	0.388
Uncharacterized conserved phage related protein	Common	0.362	0.728	RNA polymerase sigma factor	Common	0.421	0.048
undecaprenyl diphosphate synthase	Common	0.361	0.149	porin, major outer membrane protein P.I	Common	0.377	0.256
type IV pilin structural subunit	Common	0.360	0.761	fimbrial protein MS11-D1 precursor	Common	0.375	0.551
phaseolotoxin-insensitive ornithine carbamoyltransferase	Common	0.356	0.146	ABC transporter-like protein	Common	0.333	0.215
glucose-6-phosphate isomerase	Common	0.349	0.137	undecaprenyl diphosphate synthase	Common	0.328	0.051
drugs-transport transmembrane ATP-binding protein ABC transporter	Common	0.338	0.270	immunogenic lipoprotein A	Common	0.326	0.175
alkyl hydroperoxide reductase C protein AhpC	Common	0.331	0.191	nucleoside diphosphate kinase	Common	0.321	0.077
macrophage infectivity potentiator	Common	0.309	0.314	glucose-6-phosphate isomerase	Common	0.318	0.039

synthase II	Common	0.285	0.047	serine/threonine protein kinase	Common	0.316	0.070
unknown	Common	0.279	0.184	Uncharacterized conserved phage related protein	Common	0.291	0.259
(3R)-hydroxymyristoyl ACP dehydratase	Common	0.273	0.134	outer membrane protein A	Common	0.283	0.143
flagellin subunit	Common	0.273	0.159	dTDP-glucose 4,6-dehydratase	Common	0.283	0.091
dTDP-glucose 4,6-dehydratase	Common	0.260	0.101	Porphobilinogen synthase	Common	0.280	0.112
two component transcriptional regulator, winged helix family	Common	0.252	0.129	histidine transport system permease protein HisQ	Common	0.273	0.098
nitrate reductase subunit alpha	Common	0.252	0.077	macrophage infectivity potentiator	Common	0.268	0.025
outer membrane protein A	Common	0.248	0.174	invasion associated secreted endopeptidase	Common	0.267	0.375
glutamine synthetase, type I	Common	0.247	0.114	synthase II	Common	0.266	0.121
Dot/Icm type IV secretion system effector LirB	Common	0.236	0.152	alkyl hydroperoxide reductase	Common	0.260	0.197
Collagen-binding protein A	Common	0.232	0.301	trifunctional thioredoxin/methionine sulfoxide reductase A/B protein	Common	0.257	0.163
UDP-galactopyranose mutase	Common	0.229	0.085	glutamine synthetase	Common	0.250	0.115

Table S8. Rank distribution of the top 50 MvirDB functions assigned for the mRNA reads (relative abundance per participant).

CLP				Control			
**Function**	**Group-specificity**	**Mean**	**Standard deviation**	**Function**	**Group-specificity**	**Mean**	**Standard deviation**
translation initiation factor IF-1	Common	4.347	1.396	translation initiation factor IF-1	Common	2.200	0.852
50S ribosomal protein L13	Common	2.798	0.678	pyruvate formate lyase I	Common	1.939	0.722
RecName: Full=50S ribosomal protein L6;	Common	2.158	0.915	hypothetical protein	Common	1.758	0.879
pyruvate formate lyase I	Common	1.954	0.591	50S ribosomal protein L13	Common	1.744	0.532
ABC transporter, ATP-binding protein	Common	1.355	0.272	surface-anchored fimbrial subunit	Common	1.502	1.697
surface-anchored fimbrial subunit	Common	1.191	0.816	RecName: Full=50S ribosomal protein L6;	Common	1.371	0.484
chaperonin GroEL	Common	1.026	0.616	ABC transporter, ATP-binding protein	Common	1.292	0.209
hypothetical protein	Common	1.010	0.320	ABC-type transporter, ATPase component	Common	0.969	0.496
30S ribosomal protein S9	Common	0.970	0.339	30S ribosomal protein S9	Common	0.862	0.291
ATP-dependent Clp protease proteolytic subunit	Common	0.914	0.198	ATP-dependent Clp protease proteolytic subunit	Common	0.805	0.177
fructose-bisphosphate aldolase	Common	0.834	0.475	fructose-bisphosphate aldolase	Common	0.753	0.490
DNA-directed RNA polymerase subunit beta`	Common	0.787	0.142	oligopeptide ABC transporter, ATP-binding protein	Common	0.702	0.088
molecular chaperone DnaK	Common	0.767	0.369	chaperonin GroEL	Common	0.696	0.153
DNA-directed RNA polymerase subunit beta	Common	0.761	0.058	RecName: Full=Major outer membrane protein P.IB; Short=Protein IB; Short=PIB; AltName: Full=Porin; Flags: Precursor;	Common	0.673	0.343
glutamate dehydrogenase	Common	0.728	0.288	glutamate dehydrogenase	Common	0.665	0.229
oligopeptide ABC transporter, ATP-binding protein	Common	0.716	0.122	molecular chaperone DnaK	Common	0.662	0.232
ABC-type transporter, ATPase component	Common	0.676	0.284	ABC-type transporter, permease component	Common	0.633	0.369

hypothetical protein gbs0131	Common	0.659	0.178	DNA-directed RNA polymerase subunit beta	Common	0.632	0.115
RecName: Full=Preprotein translocase subunit secY;	Common	0.639	0.151	hypothetical protein gbs0131	Common	0.603	0.178
protein found in conjugate transposon	Common	0.618	0.551	DNA-directed RNA polymerase subunit beta`	Common	0.584	0.043
30S ribosomal protein S1	Common	0.607	0.134	30S ribosomal protein S1	Common	0.577	0.168
RecName: Full=ATP-dependent Clp protease ATP-binding subunit ClpX;	Common	0.548	0.064	RecName: Full=Acetate kinase; EC=2.7.2.1; AltName: Full=Acetokinase;	Common	0.567	0.053
hypothetical protein gbs0137	Common	0.539	0.136	protein found in conjugate transposon	Common	0.562	0.378
thioredoxin reductase	Common	0.539	0.137	RecName: Full=Outer membrane protein P.III; AltName: Full=Gonococcal protein III; Short=PIII; Flags: Precursor;	Common	0.521	0.150
pyruvate kinase	Common	0.505	0.198	hypothetical protein gbs0130	Common	0.510	0.487
RecName: Full=Acetate kinase; EC=2.7.2.1; AltName: Full=Acetokinase;	Common	0.498	0.089	putative ABC transporter ATP-binding protein	Common	0.509	0.092
RecName: Full=Protein translocase subunit SecY;	Common	0.497	0.148	ABC-type transporter, periplasmic component	Common	0.508	0.427
glucose-1-phosphate thymidylyltransferase	Common	0.495	0.190	ornithine carbamoyltransferase 2, chain F; CP4-6 prophage	Common	0.501	0.159
alkyl hydroperoxide reductase subunit C	Common	0.486	0.284	triosephosphate isomerase	Common	0.498	0.126
30S ribosomal protein S15	Common	0.452	0.157	RecName: Full=ATP-dependent Clp protease ATP-binding subunit ClpX;	Common	0.491	0.080
tetracycline resistance protein	Common	0.451	0.184	RecName: Full=Preprotein translocase subunit secY;	Common	0.460	0.103
polynucleotide phosphorylase/polyadenylase	Common	0.446	0.051	thioredoxin reductase	Common	0.453	0.163

ABC-type transporter, permease component	Common	0.425	0.213	glucose-1-phosphate thymidylyltransferase	Common	0.447	0.059
putative ABC transporter ATP-binding protein	Common	0.417	0.128	pyruvate kinase	Common	0.423	0.200
glutamine synthetase GLNA1 glutamine synthase GS-I	Common	0.413	0.105	P60 extracellular protein, invasion associated protein Iap	Common	0.422	0.615
RecName: Full=Outer membrane protein P.III; AltName: Full=Gonococcal protein III; Short=PIII; Flags: Precursor;	Common	0.403	0.484	thioredoxin	Common	0.410	0.156
ABC transporter, permease protein	Common	0.399	0.166	ribose-phosphate pyrophosphokinase	Common	0.377	0.058
hypothetical protein gbs0141	Common	0.387	0.205	IS1480 transposase	Common	0.371	0.467
endopeptidase Clp ATP-binding chain C	Common	0.376	0.096	RecName: Full=Protein translocase subunit SecY;	Common	0.354	0.061
SubName: Full=ClpB protein;	Common	0.375	0.175	30S ribosomal protein S15	Common	0.353	0.179
hypothetical protein lin0097	Common	0.372	0.144	alkyl hydroperoxide reductase subunit C	Common	0.353	0.390
pyruvate formate lyase-activating enzyme 1	Common	0.366	0.078	phosphoserine aminotransferase	Common	0.346	0.124
ornithine carbamoyltransferase 2, chain F; CP4-6 prophage	Common	0.366	0.141	methylmalonyll-CoA mutase	Common	0.345	0.093
thioredoxin	Common	0.350	0.196	SubName: Full=ClpB protein;	Common	0.341	0.083
RecName: Full=Major outer membrane protein P.IB; Short=Protein IB; Short=PIB; AltName: Full=Porin; Flags: Precursor;	Common	0.347	0.357	cold shock protein CspC	Common	0.341	0.374
ribose-phosphate pyrophosphokinase	Common	0.329	0.071	hypothetical protein gbs0137	Common	0.333	0.132
hypothetical protein SAG0145	Common	0.326	0.103	ABC transporter, permease protein	Common	0.331	0.058
putative lysil-tRNA synthetase LysU	Common	0.320	0.087	ribose ABC transporter ATP-binding protein	Common	0.330	0.086
RNA polymerase sigma factor	Common	0.318	0.091	seryl-tRNA synthetase	Common	0.329	0.110
triosephosphate isomerase	Common	0.313	0.066	polynucleotide	Common	0.323	0.059

phosphorylase/polyadenylase
